# Physiological responses to proposals during dyadic decision-making conversations

**DOI:** 10.1371/journal.pone.0244929

**Published:** 2021-01-22

**Authors:** Melisa Stevanovic, Samuel Tuhkanen, Milla Järvensivu, Emmi Koskinen, Enikö Savander, Kaisa Valkia

**Affiliations:** 1 Faculty of Social Sciences, Tampere University, Tampere, Finland; 2 Department of Digital Humanities, University of Helsinki, Helsinki, Finland; 3 Faculty of Social Sciences, University of Helsinki, Helsinki, Finland; 4 Department of Psychiatry, Päijät-Häme Central Hospital, Lahti, Finland; The Hong Kong Polytechnic University, HONG KONG

## Abstract

A novel conversation-analytically informed paradigm was used to examine how joint decision-making interaction, with its various types of proposal sequences, is reflected in the physiological responses of participants. Two types of dyads–dyads with one depressed and one non-depressed participant (N = 15) and dyads with two non-depressed participants (N = 15)–engaged in a series of conversational joint decision-making tasks, during which we measured their skin conductance (SC) responses. We found that the participants’ SC response rates were higher and more synchronized during proposal sequences than elsewhere in the conversation. Furthermore, SC response rates were higher when the participant was in the role of a proposal speaker (vs. a proposal recipient), and making a proposal was associated with higher SC response rates for participants with depression (vs. participants without depression). Moreover, the SC response rates in the proposal speaker were higher when the recipient accepted (vs. not accepted) the proposal. We interpret this finding with reference to accepting responses suggesting a commitment to future action, for which the proposal speaker may feel specifically responsible for. A better understanding of the physiological underpinnings of joint decision-making interaction may help improve democratic practices in contexts where certain individuals experience challenges in this regard.

## Introduction

During the most recent years, the field of empirical social interaction studies has witnessed a rise of novel interdisciplinary research approaches. A central role in this regard has been played by conversation analysts who started to show interest in the study of emotion (see e.g., [1]). In addition to describing the details of how various lexical, prosodic, and facial expression of emotion are embedded in, and regulated by, the sequential structures of social interaction, there have been attempts to consider the psychophysiological underpinnings of social interactional phenomena in a level of detail that has been unprecedented in the past [2, 3]. This line of research has also opened a fresh avenue to also consider those asymmetries of interactions that may be attributed to participants’ specific clinical and neurological conditions, such as autism spectrum disorder [4]. This paper presents a contribution to this emerging line of research. Here, we seek to enhance understanding of how joint decision-making interaction with its different types of proposal sequences is reflected in the physiological responses of participants with and without depression.

### Physiological underpinnings of social interaction

There is much psychophysiological research on individuals’ reactions to emotional stimuli [5]. Human autonomic nervous system consists of two components: parasympathetic and sympathetic, which work together to regulate physiological arousal and other body functions [6]. While the parasympathetic nervous system works to maintain homeostasis and resting state of the body, the sympathetic nervous system primes the body for action in stressful situations. Sympathetic nervous system response, which this study focuses on, can lead to physiological effects such as increased heart rate, increased respiratory rate and increased secretion in the sweat glands [7]. Sweating in turn increases the electrical conductivity of the skin. Skin conductance (SC) response has therefore been often used as a measure of emotional arousal, and increased activity has been linked to stimuli meant to elicit feelings of disgust, threat, or fear, but also to pleasant or happy stimuli [8–11].

Social interaction is a primordial, naturally occurring locus for emotional stimuli. Still, previous research in this field has been scarce and fragmentary. Still, two basic ways in how the topic has been addressed in the past may be identified. First, a set of studies has focused on how events of social interaction relate to an *increase* or *decrease* in the indicators of participants’ emotional arousal [2, 3, 12–14]. For example, focusing on storytelling and story reception, Peräkylä and colleagues [3] found that an increased level of affiliative story reception is associated with a decrease in the storyteller’s arousal and an increase in the story recipient’s arousal, as indicated in the participants’ SC response during the storytelling episodes (see also [4]). Second, there are studies on the momentary *similarity* (e.g., synchronicity or correlation) in the physiological changes in the participants interacting with each other [15–18]. In these studies, physiological synchronicity has been linked to both positively and negatively valenced affective interactional events, which has led researchers to conclude that such synchronicity is a feature of intense social interaction, which may range from competitive computer games [17] to fire-walking rituals [18].

While many studies on the physiological underpinnings of social interaction have operated within a relatively gross time frame (see e.g., [12, 15]), it is important to note that physiological indices of arousal provide meaningful information only with reference to specific behavioral correlates. A controlled investigation of how various spontaneously produced interactional behaviors, such as various linguistic or embodied communicative expressions, have certain affective consequences in specific interactional contexts, is a challenging endeavor and this type of research is still in its infancy. Recent key advances in the field include studies on the affective implications of metaphor use. Drawing on data from picture-based counseling practice, Tay and colleagues [12] found that participants who were prompted by the counselor towards the symbolic interpretation of pictures were physiologically more aroused than those who were prompted towards literal interpretation, as indicated by the participants’ skin conductance responses during the counseling interaction. Similar results were also obtained in another study [13], in which the use of a metaphorical speaking style in response to a question about academic problems, was associated with increased affective engagement.

In a similar vein, conversation analytic studies on social interaction have highlighted the need to identify those very events of interaction that underlie participants’ physiological responses. While conversation analysis is specifically about how adjacent conversational actions are chained in sequences of adjacent turns produced by different participants, it has drawn attention to the foundational experiences of raising expectations and having them responded to in specific ways. Indeed, having such expectations fulfilled or frustrated is what provides for a continuous emotional substrate for our everyday social-interactional encounters. A better understanding of these micro-experiences is thus fundamental for social, behavioral, and psychological sciences–it is basically our actions and how these are responded to that underlie the emergence of both our identities and those social structures (e.g., social relationships) that we are part of [19]. In this paper, we seek to shed light on these micro-experiences in the context of joint decision-making interaction and its constituent conversational actions: proposals and their responses.

### Joint decision-making in dyadic interaction

Conversation analytic research on joint decision-making has shown it to be a complex and challenging interactional endeavor ([20–28], even in a dyad where complex group dynamics do not need to be taken into consideration [29–34]. Joint decision-making involves both exertion of control over the content of interaction and flexible responses to the co-participant’s analogous attempts. All this necessitates the mastery of a wide range of interactional practices, such as regulating one’s syntax, lexical choices, prosody, body postures, and gaze, which may be used strategically either to keep a joint decision-making sequence alive or to abandon it. The core of a joint decision-making sequence consists of the “adjacency pair” [35] that starts with a *proposal* and makes relevant an *acceptance* as a preferred response, although the sequence gets frequently expanded as the participants commonly fail to come up with proposals that their co-participants can immediately accept. The mere act of making a proposal involves a high level of agency: it necessitates that a participant puts something of themselves “out there” for the co-participant to judge. Furthermore, a proposal is a powerful conversational action, which entails, not only a claim of the right to have a word to say in the matter at hand, but also a claim of the right to determine the content of the participants’ local interactional agenda. Proposal speakers have been shown to be sensitive to these implicit claims, orienting to a need to mitigate them in various ways [32, 33]. What is not yet known, however, is whether the making of a proposal is also associated with a higher level of arousal, compared to other constituent actions of joint decision-making interaction.

**Fig 1:**
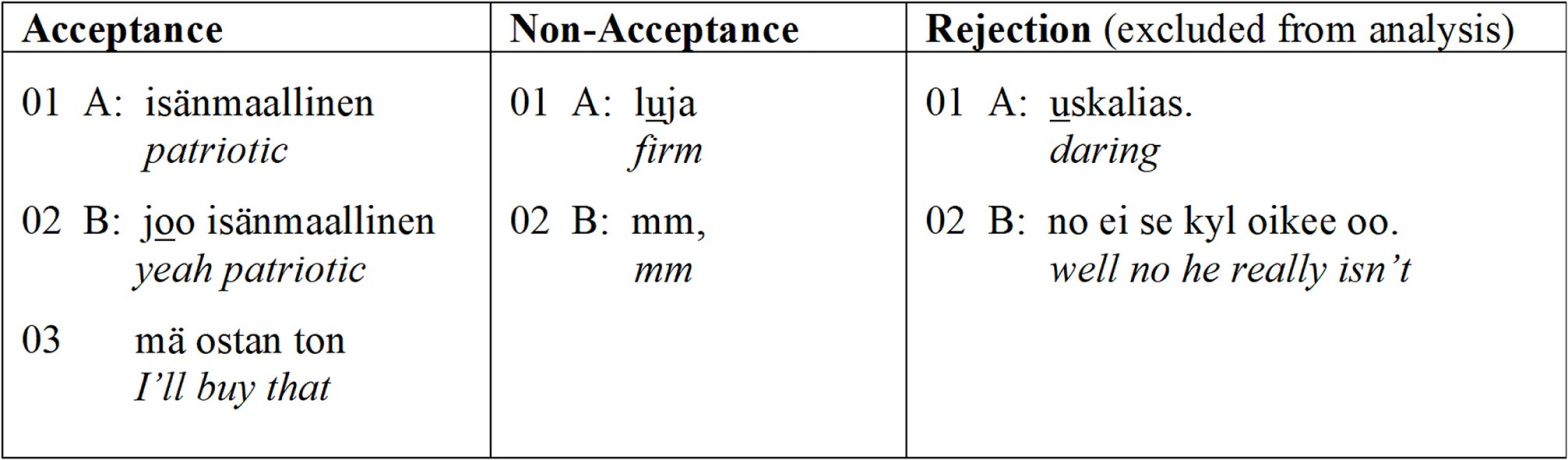
Examples of proposal sequences. Proposal sequences with different outcomes: acceptance, non-acceptance, and rejection.

A proposal is not yet a decision. Thus, as has been repeatedly pointed out in conversation-analytic studies on joint decision-making, it is essentially in and through the recipients’ subsequent responses to the proposals that joint decisions emerge [29, 36]. Accepting responses have the capacity to steer the conversation toward a joint decision, which becomes established when the participants publicly display their commitment to a specific choice of action (e.g., A: “Let’s take it.” B: “Yes, let’s take it.” [30]). Rejecting responses, in contrast, are commonly oriented to as problematic and thus also avoided [37, 38]. In this vein, Stevanovic [30] has suggested that the basic mechanism of refraining from accepting a proposal is to abandon it before the participants have established their commitment to it. Such non-acceptances can consist of silences or minimal response tokens that display recipiency and attention. The proposal recipients may even display their in-principle acceptance of the proposal speaker’s idea, but still the missing “decision component” of the sequence leads to a *de facto* rejection of a proposal. Thus, due to the participants’ ways of generally avoiding explicit rejections, the recipients’ most common ways of treating proposals are basically of two types: acceptances and non-acceptances (for examples from our dyadic decision-making experiment, where proposals typically take the form of single adjectives, see Fig 1).

No research thus far has examined the physiological and affective underpinnings of accepting vs. non-accepting responses to proposals. An accepting response by the co-participant could be hypothesized to function analogously to the affiliative responses to storytelling, which have the capacity to physiologically “calm down” the storyteller [3]. In this case, an accepting response to a proposal would calm down the proposal speaker, who might feel vulnerable and uneasy about how the co-participant sees the reasonability and feasibility of his or her proposal (on the vulnerability of self to social interaction, see [39, 40]). But then again, telling a story and making a proposal are very different types of social actions. First, they entail dissimilar degrees of projection for upcoming actions: if a proposal is not considered as relevant for the participants’ current activity, it can be abandoned by not pursuing it anymore [30], whereas an initiation of a story involves a speaker reserving for him- or herself the conversational floor for multiple turns at talk and others expecting that what is being told is worth telling [41–44]. Second, while storytelling is basically about sharing experiences [45], joint decision-making involves “a commitment to future action” [26 p. 70], for which the proposal speaker may feel specifically responsible for. From this point of view, the physiological consequences of accepting responses to proposals could, in contrast to the affiliative responses to storytelling, lead to an increased level of arousal in the proposal speaker.

In this paper, we consider how participants’ physiological responses during joint decision-making conversations are related to the local sequential context of interaction–that is, to the specific actions that constitute the joint decision-making activity (i.e., proposals and their responses). A deeper understanding of these processes could help shed light on the specific vulnerabilities that certain individuals might experience when being involved in joint decision-making [46]. Furthermore, knowledge about the emotional and physiological “cost” of making proposals would increase understanding about the challenges that different individuals may have with respect to participation in micro-level democratic practices and decision-making encounters.

### Depression, decision-making, and social interaction

Major depressive disorder (MDD), commonly known as *depression*, is one of the most prevalent and debilitating forms of psychopathology [47]. One of the characteristic symptoms of depression listed in the Diagnostic and Statistical Manual of Mental Disorders [48] involves difficulties in decision-making. Studies have shown that participants with depression not only have difficulties in making decisions [49, 50], but that also those decisions that they eventually make can follow atypical patterns [51–53]. For example, due to their altered sensitivity to positive and negative social cues, such as rewards and punishments [54–56] individuals with depressive symptoms may be more conservative when making decisions about potentially rewarding outcomes [57], have difficulties perceiving when a previously bad contingency has become good [51], and tend to overestimate the risks and underestimate the benefits associated with specific decisions [e.g., 58]. While such atypical patterns have thus far been identified in the context of individual decision-making, it is largely an open question whether depression leads to atypical behavioral patterns also in social interactional contexts, where real-life decisions are typically done.

Depression affects people’s communication behaviors, which, in turn, influence their social relationships [59–63]. This pattern has been specifically highlighted in research within the framework of the interpersonal or interactional theory of depression [64, 65], which has shown that individuals with depression may exhibit aversive communication behaviors such as avoidance of conversations that may make one feel rejected, criticized, or disappointed [66] and excessive reassurance seeking, hostility, and demands [67]. This line of research has, for example, pointed to a linkage between depressive symptoms and relationship dissatisfaction [68–71], while this linkage, again, has been found to be mediated by communication behaviors such as less self-disclosure and more destructive conflict management strategies by the partner [72] and disengaged couple communication [73]. Furthermore, the inverse relation between the number of depressive symptoms and relationship quality has been shown to be more relevant for women than for men [74].

Despite the significant and extensive bodies of research on communication behaviors associated with depression, not much is known about how exactly depression shows in the turn-by-turn unfolding of sequences of social interaction. Research on narratives and storytelling in therapeutic interactions and clinical interviews has however pointed to a specific lack of agency in the depression-related patterns of language use, which we consider potentially relevant for the present considerations. For example, individuals with depression have difficulties disclosing specific details of their life circumstances, tending instead to produce overgeneral autobiographical narratives that contain expressions of helplessness and low personal agency [75, 76]. Similarly, conversation analytic studies of psychotherapy sessions have highlighted the element of a lack of control in the talk of individuals with depression [77, 78]. Research on treatment-decision making in the context of mental health care has repeatedly pointed to difficulties that physicians have in involving their patients in making decisions about their own treatment [79–82]. It is therefore possible that a depression-related sense of a lack of agency may show specifically in the details of joint decision-making sequences.

Social interaction is not only about language use, but it is also a locus of fundamentally embodied emotional interchanges, where behavioral and physiological aspects of experience are deeply intertwined. Such processes may vary with respect to gender. For example, women have been shown to be physiologically more reactive to social stressors than men [83], their greater stress responses possibly contributing to the increased rates of depressive symptoms in women [84]. However, there is a lack of consensus on how depression itself influences physiological reactions. In general, depression is associated with dysregulation in both parasympathetic and sympathetic branches of the autonomic nervous system (ANS), which has been demonstrated in several studies [85–91]. On the one hand, these idiosyncrasies include a flat or low SC profile [92], which seems to be a reliable feature of depression and a valid marker of suicidal risk [90] and is consistent with early theories of emotion in depression, which have emphasized the behavioral and physiological underarousal as a prominent part of depressive symptomatology [93, 94]. On the other hand, depression has been connected to pathological worry and increased threat-arousal responses [95, 96]. In addition, a depression-induced lack of coordinated interpersonal connection has been suggested to decrease the degree of psychophysiological synchronicity between the participants [97, 98]. In this study, we provide a preliminary exploration of how female participants with and without depression respond physiologically to a set of very specific potential “social stressors”, which consist of making and responding to proposals in joint decision-making interactions.

While reaching genuinely joint decisions is always a challenging endeavor [30], the understanding of the physiological responses underlying the micro-experiences of producing and responding to proposals may enhance understanding of these challenges. Such knowledge could then inform the development of more effective democratic practices in contexts such as social and health care [99–101], where it is not only that participants might have specific challenges in this regard but where the decisions are also likely to be of particular importance for the same participants’ physical and mental well-being.

### Research question and hypotheses

This study is guided by one main research question: How are the different types of decision-making sequences reflected in the physiological responses in interacting participants? In addition, we want to explore the possibility that these responses would differ for participants with and without depression. The more specific hypothesis, which we seek to test empirically, are the following:

*Hypothesis 1*: SC response rates are dependent on the local sequential context of interaction, being higher during proposal sequences than during non-proposal sequences.
*Hypothesis 2*: Degree of SC synchronicity is also dependent on the local sequential context of interaction, with proposal sequences leading to higher synchronicity than non-proposal sequences.
*Hypothesis 3*: SC response rates will be higher when a participant herself produces a proposal, as compared to being produced by a recipient of a co-participant’s proposal.
*Hypothesis 4*: Behavioral responses to proposals are reflected in the participants’ physiological responses

1. Accepting responses to proposals, as opposed to non-accepting responses, are associated with lower SC response rates in the proposer.
2. The emergence of a joint decision may be an arousing interactional event in itself and may thus lead to higher SC response rates in the proposer.

Due to the physiological idiosyncrasies associated with depressive symptomatology, SC responses to proposal sequences could be different for depressed vs. non-depressed participants, while the SC synchronicity could be lower for dyads with depressed participants vs. non-depressed participants. We will explore these possibilities, as well.

## Methods

### Participants

Two types of female adult participants were recruited: participants (N = 45) who had not got a depression diagnosis within the past ten years and participants (N = 15) who had been diagnosed with middle stage depression within the past 12 months. As a proxy for controlling for similar general cognitive capacities in both participant groups, only participants who had at least five years (or three years if under 25) working life experience and at least one bachelor’s degree (or equivalent number of university-level studies) were recruited for the study. The participants (N = 60) were divided into two groups of pairs: 15 pairs, where one participant had a depression diagnosis (“case pair”), and 15 pairs, where neither participant had been diagnosed with depression within the past ten years (“comparison pair”).

In both cases, the participants’ recruitment started with an announcement published in the social media. Those who responded to the announcement were called by the phone and asked for background information (age, education, work history, information on earlier depression diagnosis). Based on this information the candidate was either excluded from the research or guided to the group of participants with depression diagnosis (N = 15) or to the comparison group of participants without any depression diagnosis (N = 45). The clinical status of participants with middle stage depression diagnosis was confirmed by a medical specialist in psychiatry and general practice, who met each participant privately and did a clinical interview and needed inquiry on symptoms (Beck Depression Inventory (BDI) and Montgomery–Samberg Depression Rating Scale (MARDS)). The medical specialist also took care of the arrangement for the needed care for the participants after the research had ended.

Before the experiment both participants were guided to fill out a set of questionnaires and the purpose of the research was clarified verbally and in writing. The participants were told that, in this research, we will study structures of interaction in decision-making and the impact of mood on the dynamics on joint-decision-making. The clinical status of those participants who had a diagnosis of depression was not revealed to the interaction partner, because the information could have affected the dynamics of the subject of study and, furthermore, could have unnecessarily stigmatized the participants with depression. At this point the participants were also given the opportunity to ask questions about the research. The participants were informed about the researcher’s obligation to maintain secrecy, the practices of the anonymity and data management, the publication of the research results and the voluntary participation in the research. It was also clarified, that even after the written, consciously given consent the participant would be allowed to make his/her consent reversible at any time of the research project without this affecting the position or treatment of the participant. The participants were also told how to reverse their consent in practice.

### Ethics

Participation in the project was voluntary. All participants gave their written consent to the study after having been informed about the aims of the study and about their rights to withdraw their consent anytime they wished. Institutional Review Board approval was obtained from the Ethics Committee of the Helsinki University Central Hospital [18.06.2018].

### Equipment

NeXus-10 (Mind Media, Netherlands) devices were used to measure electrodermal activity/skin conductance (SC) and blood volume pulse (BVP) from both participants at a 128 Hz sampling rate. SC was measured with two foam electrodes that were placed on the medial side of the left foot. The BVP sensor was attached to the second digit of the left foot. Eye-movements were recorded at 60 Hz sampling rate with two binocular head-mounted Pupil Labs eyetrackers (Pupil Labs UG haftungsbeschrnkt, Berlin, Germany). The eye-trackers were calibrated on a LG OLED55C7V 55" monitor with 16 calibration markers. The open-source Pupil Capture software (v1.8 from: https://github.com/pupil-labs/pupil) was used to record and calibrate the eye tracker. Shimmer3 IMUs (Shimmer Sensing, Ireland, Dublin) were attached to each participant’s right wrist to record linear acceleration and angular velocity. A custom-made software (https://github.com/samtuhka/InteractionExperiment-Controller) was used to synchronize the NeXus, Shimmer3 and Pupil data with Unix timestamps. Only skin conductance data was analyzed in this paper.

The participants sat facing each other at about an 120° angle from each other. The angle was chosen so that the participants would not have to change position to calibrate the eye-tracker.

### Experiment

A single dyad was studied at a time. The design of our experiment was derived from the novel experimental paradigm that we had designed in our previous study [34]. Here, the participants were asked to choose together an adjective that would best describe a target. The adjective needed to start with a given letter, and once a decision was reached, the dyad had to move to the next letter in the alphabet, deciding altogether on 8 adjectives. The task was performed twice. In one trial (consisting of 8 decisions), the adjective target was Donald Duck, and in the other, Finnish President Sauli Niinistö, while the letters were either [H, I, J, K, L, M, N, O] or [N, O, P, R, S, T, U, V]. The creation of the task was guided by the goal of trying to generate social interaction patterns that would be maximally like those that we had previously encountered in our qualitative analysis of naturally occurring planning interactions at the workplace [30–33]. As a motivation for the task, the participants were told to imagine being editors of a children’s book, teaching the alphabet to kids by featuring the target character, and they would need to choose suitable adjectives for that purpose. The type of the target (Donald Duck or President Sauli Niinistö) and the alphabet list, as well as the order of this task in relation to another task not reported here, were counterbalanced across pairs.

After the participants were given the permission to start the task, their conversation was to unfold freely. In other words, just like in any everyday interactions where two people need to make a series of routine, low-stake decisions together (e.g., agreeing on a dinner menu or choosing songs to be played at a party), the participants had to organize their interactions in by themselves–in reliance of all the mundane interactional practices by which new items of decision-making are brought into joint focus of attention, proposals are accepted or turned down, decision are established, and transitions from one joint decision-making sequence to a next are achieved (for the unfolding of a typical trajectory of conversation with reference to one letter item of decision making, see S1 Appendix).

At the beginning of each session, the participants filled in the following questionnaires: (1) Locus of Control Scale [102], (2) Self-Monitoring Scale [103], (3) Empowerment Scale [104] (4) Ten-Item Personality Inventory, TIPI [105], as well as a set of questions from our previous studies [34], which targeted the participants’ perceptions and experiences of the task requirements, their interaction partner, and the dynamics of interaction.

### Annotations

We used Praat [106] to annotate all proposals from the interactions based on the audio recordings. In each of the two trials, the dyad performed eight decision-making tasks in a row, each of which contained at least one “proposal” and one” response” to it (mean: 2.2 proposals for a letter). In all, we identified 1046 proposals from all the trials in our data with 30 dyads. The recipients’ responses to the proposals were then divided into two categories, depending on whether the proposal was accepted (44% of the proposals) or not accepted (56% of the proposals). As “non-acceptance”, we considered minimal responses, which simply displayed recipiency but did not take a stance toward the content of the proposal, and situations where the proposals were followed by at least 2-second-long silence. Outright rejections were very rare (~1% of the cases), and these have not been considered in this study.

The annotation of the responses had been done independently by two raters–with one rater annotating the entire data and the other annotating a randomly chosen sample (consisting approximately 20% of the whole data set) for validation. Inter-rater reliability was assessed using the Cohen’s kappa coefficient with the result of 0.87 –indicating almost perfect degree of agreement [107].

### SC analysis

All proposal sequences (N = 1046) were subjected to SC analysis. All data processing and visualization was done using Python scripts using the SciPy, NumPy, Pandas, Statsmodels and Matplotlib third-party libraries.

**Fig 2:**
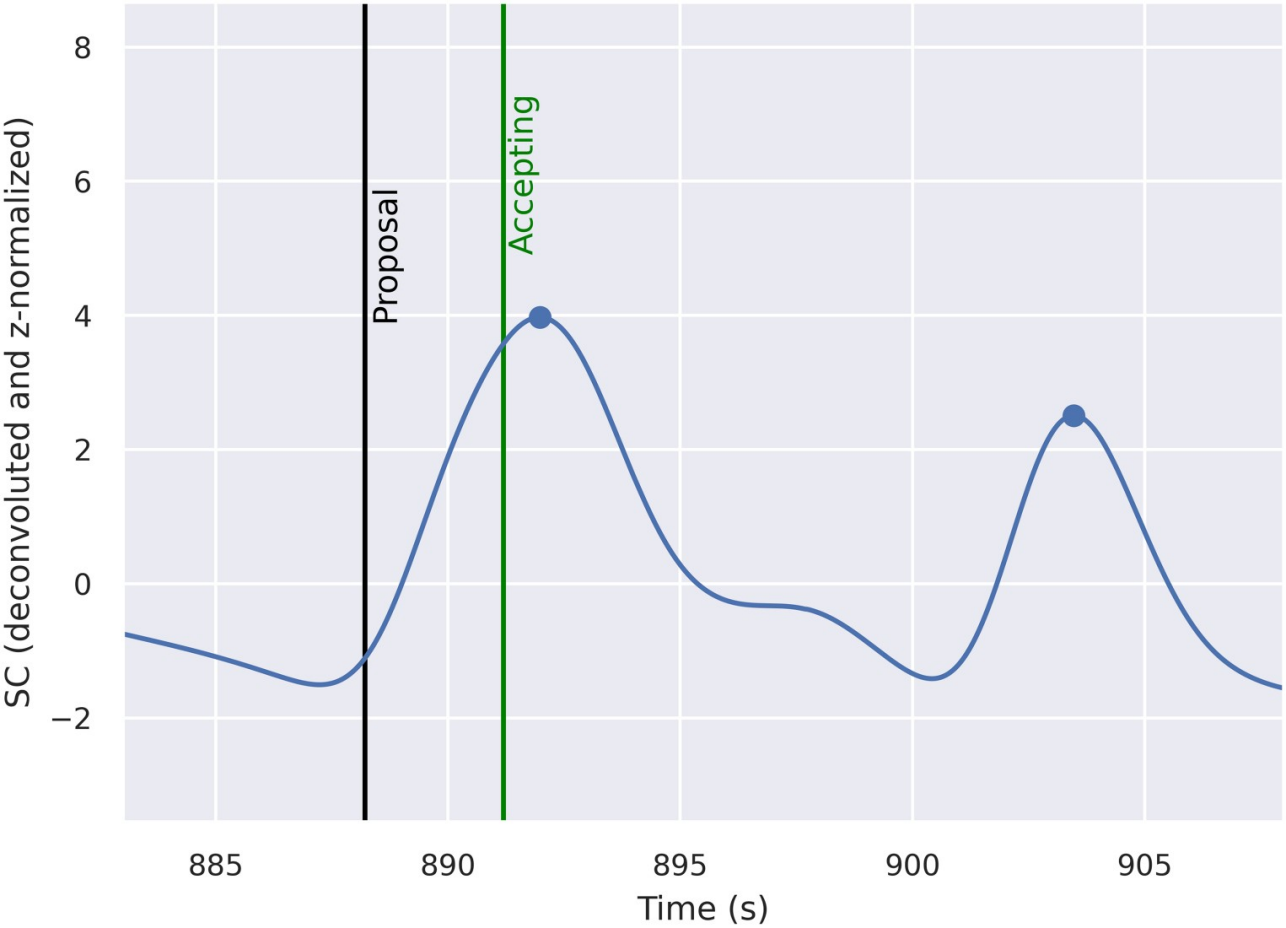
An example time series of the SC signal. The deconvoluted and z-normalized SC signal is depicted by the blue line. The blue dots indicate the peaks of individual SC responses. The black vertical line indicates the end of a proposal and the green vertical line the end of an accepting response (derived from the annotations).

To distinguish between overlapping SC responses [108, 109], the SC signal was deconvoluted using the Richardson-Lucy algorithm [110]. Individual SC responses were detected computationally through peak detection–by finding all local maxima with a prominence of at least 0.05 μS and height of at least one standard deviation above the mean level (see Fig 2). Further examples can be found in S2 Appendix.

The mean SC response rate was calculated for each proposal from one second before the end of the proposal to four seconds after the end of the proposal. The time window was chosen to both consider the fact that the participants may be able to anticipate what the proposal is about even before the proposer has finished and the fact that SC responses can be delayed by several seconds from the onset of the stimuli.

## Results

### SC response rates and synchronicity

We measured psychophysiological synchronicity by calculating Pearson correlation coefficients for each dyad between the deconvoluted SC signals of the participants of the dyad in question. The signals were synchronized with respect to time. High positive correlation would thus indicate that the participants tended to exhibit similiar of (phasic) skin conductance activity in respect to time. Furthermore, we differentiated between proposal sequences (time intervals from 1 second before a suggestion to 4 seconds after, see Methods for details) and non-proposal sequences (all other times) to examine whether the local sequential context of interaction had an effect on the degree of synchronicity.

After a Fisher transformation, the (retranformed) mean of dyad correlations during the proposal sequences was 0.20 (SD = 0.17), compared with 0.13 (SD = 0.14) during non-proposal sequences. While the correlations are very small, the differences between the proposal and non-proposal sequences are significant (dependent t-test, t = 2.51 p = 0.02, Cohen’s d = 0.47)–i.e when subtracting the correlation coefficient calculated for proposal sequences from the correlation coefficient of the non-proposal sequences for each dyad the values differ significantly from zero. There was no significant difference (dependent t-test, p = 0.68) between proposals with a non-accepting (r = 0.18) and an accepting response (r = 0.20). In terms of the SC response rates, the mean response rate of the dyads (mean of the dyad means) over the entire task was 2.76 (SD = 0.79) SC responses per minute, as compared to 4.03 (SD = 1.61) SC responses per minute during the proposal sequences (dependent t-test, t = 6.0, p < 0.001, Cohen’s d = 1.0). This suggests that the proposals and the following responses increased the overall arousal in at least one of the participants of a dyad.

With respect to the proposal sequences, there was no significant difference between the mean correlations of the depression dyads (r = 0.21) and the control dyads (r = 0.20), nor in the SC response rates (4.15 SC responses per minute in the depression dyads vs. 3.9088 in the control dyads). The mean correlation over the non-proposal sequences was slightly higher in the non-depression dyads (r = 0.16, as compared to r = 0.10), but the difference was not significant (p = 0.11).

### Skin conductance in the proposers and recipients

We probed the differences in the level of arousal between the proposal speaker and the recipient by examining the difference in SC response rates during the proposal sequences. We further segmented the participant group based on whether they had a depression diagnosis or not, resulting in four groups: proposers with depression, proposers without depression, recipients with depression, and recipients without depression.

**Fig 3:**
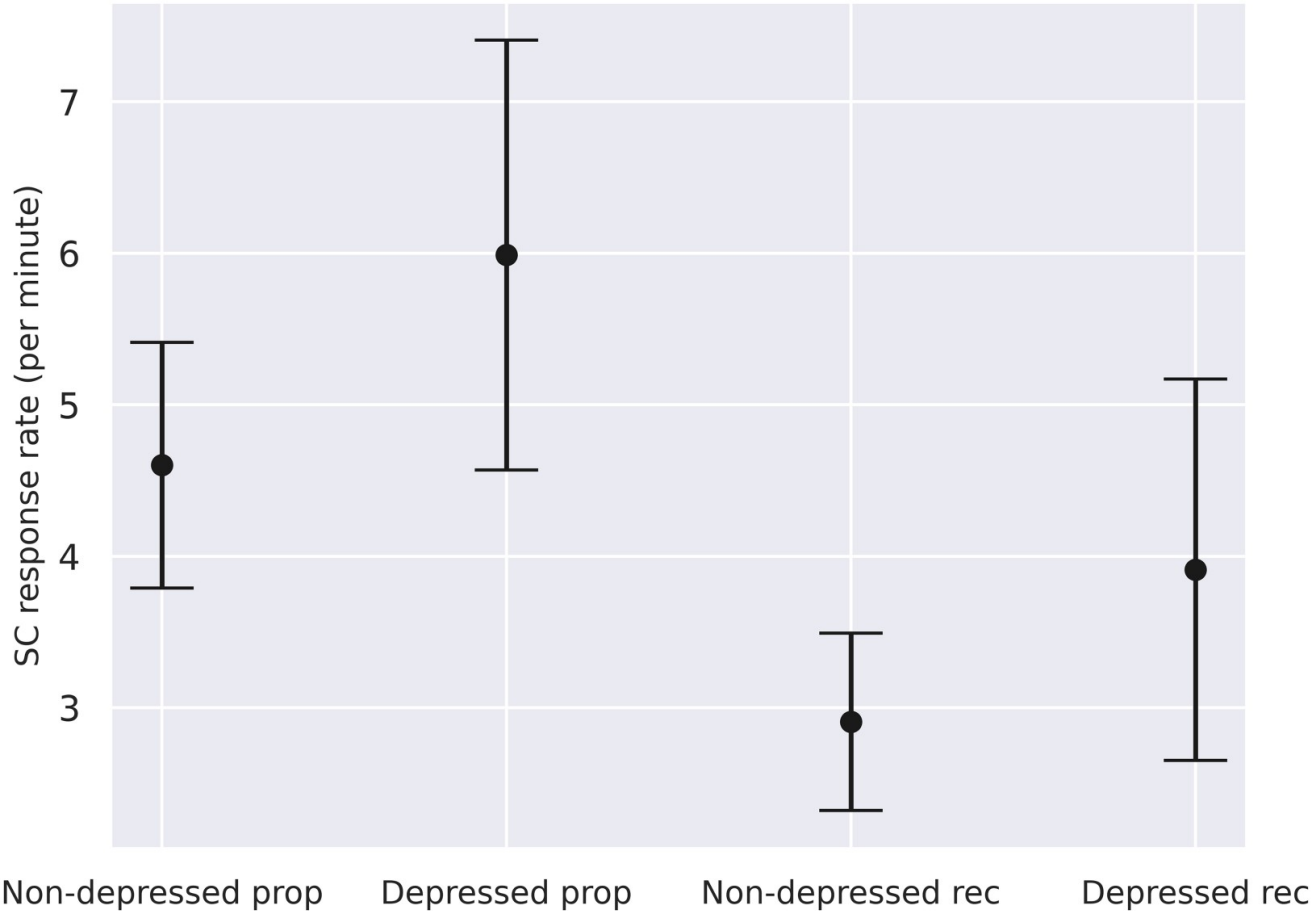
SC response rates in non-depressed and depressed proposers (prop) and recipients (rec). The black dots indicate means while the black bars represent 95% confidence intervals.

The effect of the participant’s role and depression diagnosis on their SC response rates (see Table 1 and Fig 3 for the group means) was assessed with a generalized linear mixed model (GLMM). While SC response rate was the dependent variable, depression and role (proposer or recipient) were chosen as fixed effects with the depression:role interaction term included, and dyad and participant as nested random effects (random intercepts only) with participant nested inside dyads. We chose this approach to account for the non-independence of both participants and the dyads–each participant is both a recipient and a proposer and participants of a dyad are in interaction with one another. The model summary can be seen on Table 2.

**Table 1:** Mean skin conductance responses per minute and respective standard deviations for non-depressed and depressed proposers and recipients.

	Non-depressed	Depressed
Proposer	4.60 (SD = 2.67)	5.99 (SD = 2.47)
Recipient	2.91 (SD = 1.93)	3.91 (SD = 2.20)

**Table 2:** Coefficient table of the GLMM used to assess the effect of the depression diagnosis (Diagnosis; 0 = no depression diagnosis, 1 = depression diagnosis) and the role of the participant (Role; 0 = recipient, 1 = proposer), and their interaction on the skin conductance response rates of the proposers.

	Estimate	Std. Error	df	t value	Pr(>|t|)
(Intercept)	2.8685	0.3812	58.5301	7.5253	3.645E-10 ***
Role	1.6945	0.3601	58	4.7049	1.617E-05 ***
Diagnosis	1.1559	0.6475	77.184	1.7853	0.07814
Role:Diagnosis	0.3833	0.7203	58	0.5322	0.5966

The marginal R^2^ [111] for the model was 0.17 (i.e., the fixed effects alone explain 17% of the observed variance) while the conditional R^2^ was 0.57 (i.e., fixed effects and random effects explain about 57% of the observed variance). There is no consensus on how p-values should be estimated for linear mixed models, but following the t-statistics obtained from using the Satterthwaite’s method, the effect of the participant’s role (whether proposer or recipient) was highly significant (p < 0.0001) whereas the effect of the depression diagnosis and the role:depression interaction were not.

We also examined (with a different GLMM) the SC responses in respect to the duration of the whole task and could not observe statistically significant difference (p = 0.18) in respect to the diagnosis status of the participants (2.65 responses on average per minute among participants without depression vs. 3.08 among participants with depression).

In summary, during the proposal sequences, recipients had lower SC response rates than proposers regardless of whether they had a depression diagnosis.

### Accepting vs. non-accepting responses

**Fig 4:**
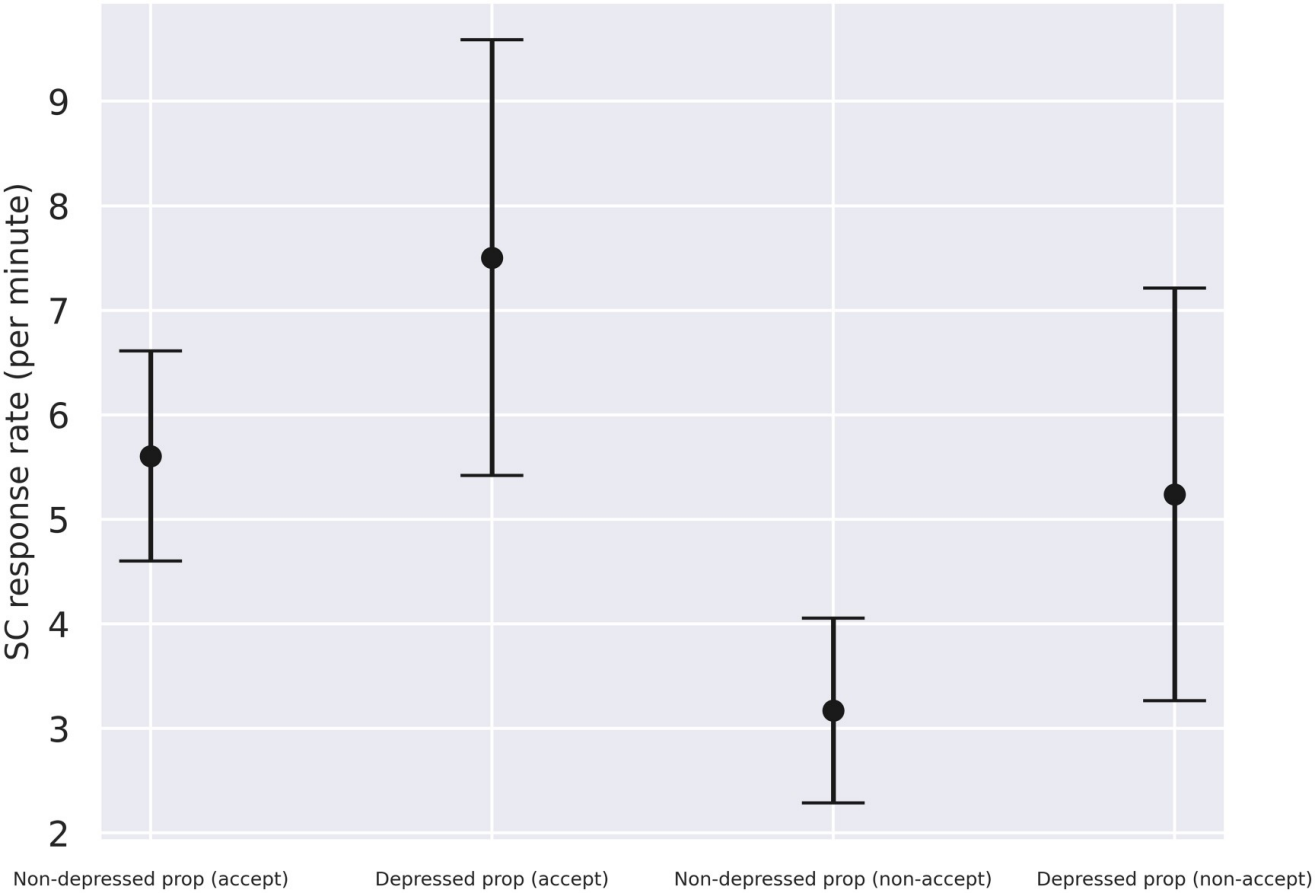
SC response rates in non-depressed and depressed proposers (prop) when the responses were accepting (accept) or non-accepting (non-accept). The black dots indicate means while the black bars represent 95% confidence intervals.

We classified responses to proposals based on whether they were accepting or non-accepting the proposal (see Methods). There was roughly the same amount of accepting responses (44%) and non-accepting responses (56%). The means and standard deviations for the four groups can be seen below (Table 3 and Fig 4).

**Table 3:** Mean SC responses per minute and respective standard deviations for the non-depressed and depressed proposers with regard to accepting and non-accepting responses.

	Accepting	Non-accepting
Non-depressed	5.61 (SD = 3.31)	3.17 (SD = 2.91)
Depressed	7.50 (SD = 3.63)	5.24 (SD = 3.44)

Similarly, to how we examined the differences between proposers and recipients, we assessed the effect of the recipient’s response on the proposer with a GLMM. Depression diagnosis and the recipient’s response (accepting/non-accepting) were chosen as fixed effects with the response:diagnosis interaction term included whereas dyad and participant were chosen as nested random effects (intercepts only). The model summary can be seen on Table 4.

**Table 4:** Coefficient table of the GLMM used to assess the effect of the depression diagnosis (Diagnosis; 0 = no depression, 1 = depression), the response type (Response; 0 = non-accepting, 1 = accepting), and their interaction on the skin conductance response rates of the proposers.

	Estimate	Std. Error	df	t value	Pr(>|t|)
(Intercept)	3.1256	0.5081	70.7293	6.1519	4.087E-08 ***
Response	2.436	0.5637	58.0002	4.3213	6.159E-05 ***
Diagnosis	2.2441	0.9493	90.2789	2.3638	0.02023 *
Response:Diagnosis	-0.1709	1.1274	58.0002	-0.1516	0.88

The marginal R^2^ for the model was 0.18 while the conditional R^2^ was 0.45. The effect of the response type (accepting vs. non-accepting) was highly significant (p < 0.0001) and the effect of the depression diagnosis was significant (p = 0.02) as well. Interestingly, accepting responses had higher response rates than non-accepting responses, and depressed proposers had higher response rates than non-depressed proposers.

## Discussion

In this study, we have considered how joint decision-making interaction with its different types of proposal sequences is reflected in the physiological responses of participants with and without depression. Overall, our hypotheses were partially supported by the empirical results, which we will discuss below.

As for *Hypothesis 1*, our data support the conclusion that SC response rates are dependent on the local sequential context of interaction, so that they are higher during proposal sequences than elsewhere in the conversation. The same pattern seems to hold also for the degree of SC synchronicity, as predicted in *Hypothesis 2*. Our results are thus consistent with earlier literature arguing that physiological synchronicity is not a matter of positivity or negativity of social interaction but a feature that accompanies periods of intense social interaction [18, 17], as our findings about the higher SC response rates during proposal sequences point to their relative intensiveness. While the synchronization of behaviors and physiological responses between two interacting participants has been an important topic during the past decades (for reviews, see [34, 112–114]), our study has demonstrated that, in addition to considering relatively long segments of interaction, such phenomena can also be addressed by adopting a relatively granular approach to the turn-by-turn sequential unfolding of interactional events.

As for *Hypothesis 3*, our data also support the conclusion that SC response rate will be higher in the role of a proposer in relation to that of the proposal recipient. A proposal is a powerful conversational act, which entails, not only a claim of the right to have a word to say in the matter at hand, but also a claim of the right to determine the content of the participants’ local interactional agenda. Proposal speakers have been shown to be sensitive to these implicit claims, orienting to a need to mitigate them in various ways [32, 33]. In making a proposal, a participant puts him- or herself into a vulnerable position, where the relevance, reasonability, and feasibility of his or her idea is to be judged by others (see [39, 40]). While the proposals speakers thus put something of themselves “out there” for others to judge, the role of the proposal recipient is quite different, involving the possibility of simply conforming to the ideas already expressed, which is an interactionally “safe” thing to do. From this point of view, it is quite understandable that the physiological underpinnings of making proposals and responding to them are quite different.

In addition to assuming that the roles of proposer and recipient are associated with different physiological response patterns, we also assumed that the different types of recipient responses to proposals are reflected in the participants’ physiological responses, as formulated in *Hypothesis 4*. However, given the scarcity of earlier literature on the topic, we presented two alternative hypotheses for the direction of the effect. First, drawing on earlier research on participants’ physiological responses to storytelling [3], we hypothesized that accepting responses to proposals function analogously to the affiliative responses to storytelling, “calming down” the storyteller (4a). However, our prediction that accepting responses to proposals, as opposed to non-accepting responses, would be associated with lower SC response rates in the proposer, does not get support from our data. Instead, the data supports our alternative hypothesis, according to which the emergence of a joint decision is an arousing interactional event and thus leads to higher SC response rates (4b). Such arousal may be accounted for with reference to proposals often being designed in ways that allow their easy abandonment [32, 33], which makes a non-acceptance of a proposal an unproblematic action to perform and receive, while an accepting response to a proposal entails “a commitment to future action” [26 p. 70], for which the proposal speaker may feel specifically responsible for.

We also explored the possibility that, due to the physiological idiosyncrasies associated with depressive symptomatology, SC responses to proposal sequences could be different for depressed vs. non-depressed participants, while the SC synchronicity could be lower for dyads with depressed participants vs. non-depressed participants. While the other differences in our data were statistically insignificant, we nevertheless found that the production of proposals is more arousing for the participants with depression than for their non-depressed comparisons. While we cannot say much about the possible mechanisms underlying this finding, one possibility is that it is connected to the earlier-mentioned phenomena of pathological worry and increased threat arousal, which have mostly been associated with anxiety (for an early study, see [95]), but which have also been shown to be a part of the etiology of depression [96], as well as to the female physiological reactivity to social stressors [74]. These phenomena, combined with the depression-related lack of agency discussed above (see [76–78]), may lead to the participants witnessing themselves contributing to potentially emerging decisions as relatively stressful. As for the existing studies reporting physiological underarousal in individuals with depression [90, 92–94], the results of our study were different. One possible explanation for the discrepancy may be the nature of our task that involved engagement in the turn-by-turn unfolding of social interaction dynamics, which differed from the previously used laboratory tasks. Our result may also have been influenced by our use of technical measurement devices (eye-trackers, electrodes, etc.), which may have been experienced as stressful by the depressed individuals. It is worth noting, though, that we found depression-related patterns of arousal only with reference to specific interactional events (the making of proposals), which suggests a lesser role for the experimental setting as a factor behind our results.

The study has several limitations, which point to a need of caution in generalizing our findings. First, all the participants in our study were female. Our results might have been different had we included male dyads or cross-gender dyads into our sample. This may hold specifically for our depression-related results, which may not have been similarly observable in males, who have been suggested to be less sensitive to social stressors than females [83]. Second, our experimental task involved talking to a stranger. Therefore, our participant sample may have been biased, favoring more socially courageous participants over the more socially shy. This may have influenced the stress-related physiological responses in all the dyads. Furthermore, since the participants in our study were strangers to each other, our behavioral and physiological results may not directly apply to interactions between everyday acquaintances, friends, or family members. Third, the sample size of depressed participants (N = 15) was quite small due to practical limitations. This limits the generalizability of our depression-related findings, which should be regarded as tentative. Fourth, the task of finding adjectives to describe a specific target, such as a cartoon character or a president requires creativity and verbal fluency, which may not be needed in many other types of joint decision-making interactions in everyday life (e.g., choosing whole or skimmed milk for the family). Finally, we may not be sure about whether and how exactly the carrying out of experimental measurements during the conversational tasks influenced the behavioral conduct of the participants in our data. As for the influence of videorecording on social interaction pattern, research in visual sociology and conversation analysis suggests that the dynamics of face-to-face social interaction are strong enough to take a precedence in the participants’ conduct even in the situations that at first come across as artificial (see e.g., [115–117]). Our research assumes that something like this also happens in interactions in experimental settings, and the subjective impressions that we get from our data largely support the assumption. In addition, though skin conductance measures provide powerful tools for assessing the level of arousal in participants, they are very general indicators of arousal and provide no direct information about the valence of that arousal. Furthermore, SC measures do not always align with self-reported arousal, and this may especially be the case in regard to clinical conditions such as depression and anxiety [118, 119].

Our study has highlighted a substantial degree of similarity in how joint decision-making interaction with its various types of proposal sequences is reflected in the bodies of participants with and without depression. However, when in the role of the proposer, our results suggest that the participants diagnosed with depression might experience moments of decision-making as more arousing than their non-depressed comparisons. A better understanding of this pattern may help to improve democratic practices in contexts that are specifically challenging for these individuals. Furthermore, our results support the overall conclusion that receiving support for your proposals is an emotionally arousing phenomenon, which might result from an increased level of stress arising from the heightened sense of responsibility for the decision or from the mere enthusiasm of having successfully contributed to a joint endeavor. Further conversation-analytically informed research on the physiological underpinnings of social interaction is needed to unravel how interactions with participants from various clinical groups and systemic features of interactional practices are intertwined.

## Supporting information

S1 Appendix (DOCX)

S2 Appendix (DOCX)
