# Supplementary material for: Physiological responses to proposals during dyadic decision-making conversations
Source: PLoS One. 2021 Jan 22;16(1):e0244929. doi: 10.1371/journal.pone.0244929 (PMC7822527; doi:10.1371/journal.pone.0244929)
Supplement: S1 Appendix — (DOCX) [file pone.0244929.s001.docx]

**Appendix A: The joint decision-making task**

The participants were asked to choose together an adjective that would best describe a target. The adjective needed to start with a given letter, and once a decision was reached, the dyad had to move to the next letter in the alphabet, deciding altogether on 8 adjectives. The task was performed twice. In one trial (consisting of 8 decisions), the adjective target was Donald Duck, and in the other, Finnish President Sauli Niinistö, while the letters were either [H, I, J, K, L, M, N, O] or [N, O, P, R, S, T, U, V]. As a motivation for the task, the participants were told to imagine being editors of a children’s book, teaching the alphabet to kids by featuring the target character, and they’d need to choose suitable adjectives for that purpose.

The conversations were to unfold freely. Still, these conversations followed a somewhat predictable pattern. First, the participants established their current task, which typically happened by the participants stating the letter with which the next adjective should begin with. Then, the participants made proposals and discussed their merits. Finally, the participants displayed commitment to one of the options previously discussed and established a joint decision (e.g., *otetaan se* “let’s take it”).

The data extract below illustrates a typical trajectory of conversational interaction generated by the task instruction. In this case, the participants try to find an adjective that would describe Donald Duck and start with the letter “P”. The transcript contains three lines of text: the Finnish original, a grammatical word-for-word gloss (for the meaning of the glossing abbreviations, see below) and a free English translation. Short silences in the conversation are indicated by “(.)”.

01 A: sit ois pee

prt be-cond letter

*then there would be ‘P’*

02 B: pöhkö

fool

*fool*

03 A: joo meinasin sanoa paksu

prt almost.do-1 say-inf thick

*yeah I almost said ‘thick’*

04 mut ei ehkä sit kuitenkaan sovi tähän tarkoitukseen

but neg perhaps prt after.all suit this-ill purpose-ill

*but I guess that after all it won’t suit this purpose*

05 (.)

06 A: mut pöhkö on aika hyvä

but fool be prt good

*but ‘fool’ is quite good*

07 A: ei tuu kyl oikein mitään muuta

neg come prt prt anything else

*nothing else really (comes to mind)*

08 B: otetaako sit se pöhkö

take-pass-q prt dem fool

*will we then take that ‘fool’*

09 (.)

10 B: tai sitte pullea tai paksu tai

or prt chubby or thick or

*or then ‘chubby’ or ‘thick’ or*

11 (.)

12 A: jotenki tekis vielä mieli löytää joku vähän erilainen

somehow do-cond prt mind find-inf some a.bit different

*somehow I would still like to find something a bit different*

13 A: miten ois paras

how be-cond best

*how about ’best’*

14 (.)

15 ((laughter))

16 A: ei se ehkä mikään paraskaan ole

neg dem perhaps any best-cli be

*no maybe he is not the best either*

17 A: parka

pitiful

*’pitiful’*

18 B: parka vois olla yks

pitiful could be-inf one

*‘pitiful’ could be one*

19 A: jos mä niiku ajattelen sitä että se on lapsille suunnattu kirja

if I prt think-1 dem-par prt it be children-all target-pppc book

*if I consider that it is book targeted to children*

20 niin mä en haluais mitään negatiivisia mielikuvia laittaa

prt I neg want-cond any negative-pl-par mental-image-pl-par put-inf

*then I would not want to out any negative mental images*

21 et joku semmonen kiva adjektiivi olis

prt some kind.of nice adjective be-cond

*so some kind of nice adjective would be (good)*

22 (.)

23 B: mikään päättäväinenkää oo tai emmä tiedä

any determined-cli be or neg+I know

*(he) isn’t determined either or I don’t know*

24 A: no voisko se olla

prt could-q dem be-inf

*well could it be*

25 (.)

26 A: kyl mä luulen et se yrittää kovasti

prt I think-1 prt dem try hard

*I do think that he is trying hard*

27 B: se vois olla

dem could be-inf

*it could be*

28 A: ei se päättäväinen aina tarkoita että onnistuis

neg dem determined always mean prt succeed-cond

*being determined does not always mean that one succeeds*

29 B: nii se on totta

prt dem be true

*yes that’s true*

30 A: et sinänsä

prt as.such

*so as such*

31 B: aikaansaava on eri juttu sit

productive be different thing prt

*being productive is a different thing*

32 A: niin (.) otetaan vaan se päättäväinen

prt take-pass prt dem determined

*yeah (.) let’s then take that ‘determined’*

33 B: nii (.) eiks aina jotain hölmöö keksi ja sit se toteuttaa

prt neg-cli always something stupid-par come.up and prt dem realize

*yeah (.) isn’t he always coming up with some stupid ideas and the he realizes (them)*

34 B: mennään sillä päättäväisellä

go-pass dem-ade determined-ade

*let’s go with that ‘determined’*

35 A: joo

prt

*yeah*

---

**Glossing abbreviations**

1, 2 person

pl plural

par partitive

ill illative

ade adessive

all allative

inf infinitive

cond conditional

cli clitic

q question clitic

neg negation

pass passive

pppc passive past participle

Singular, third person, nominative, active and present tense are forms that have been considered unmarked. These have not been glossed.
