# Supplementary material for: Physiological responses to proposals during dyadic decision-making conversations
Source: PLoS One. 2021 Jan 22;16(1):e0244929. doi: 10.1371/journal.pone.0244929 (PMC7822527; doi:10.1371/journal.pone.0244929)
Supplement: S2 Appendix — (DOCX) [file pone.0244929.s002.docx]

**Appendix B: Examples of skin conductance peak detection**

Below are four examples of what the deconvoluted skin conductance (SC) signal (blue line) and peak detection looks like. The black vertical lines indicate individual peaks – i.e., the detected SC responses. Each figure displays a 4-minute segment from the experiment.

**
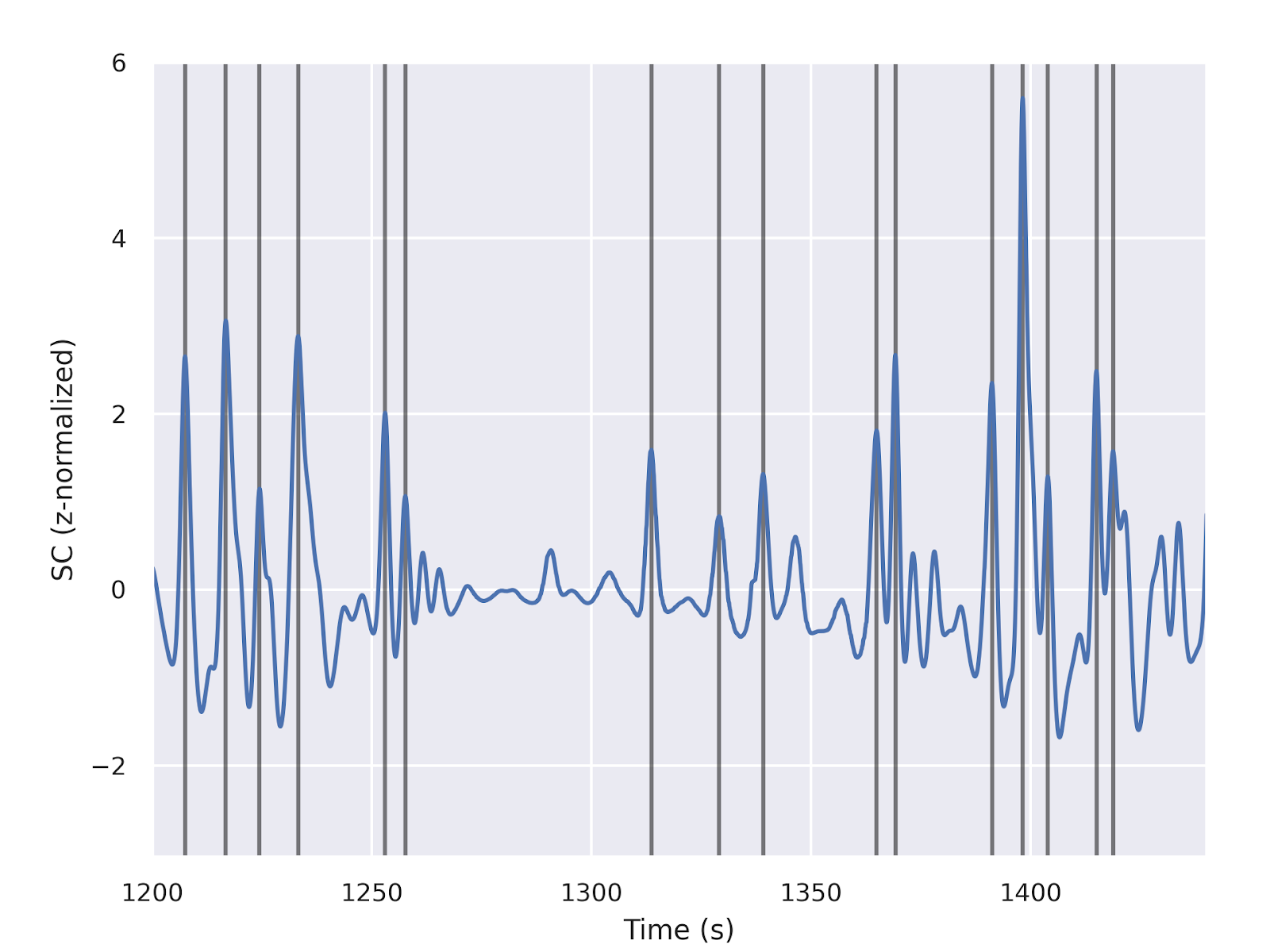
**

**
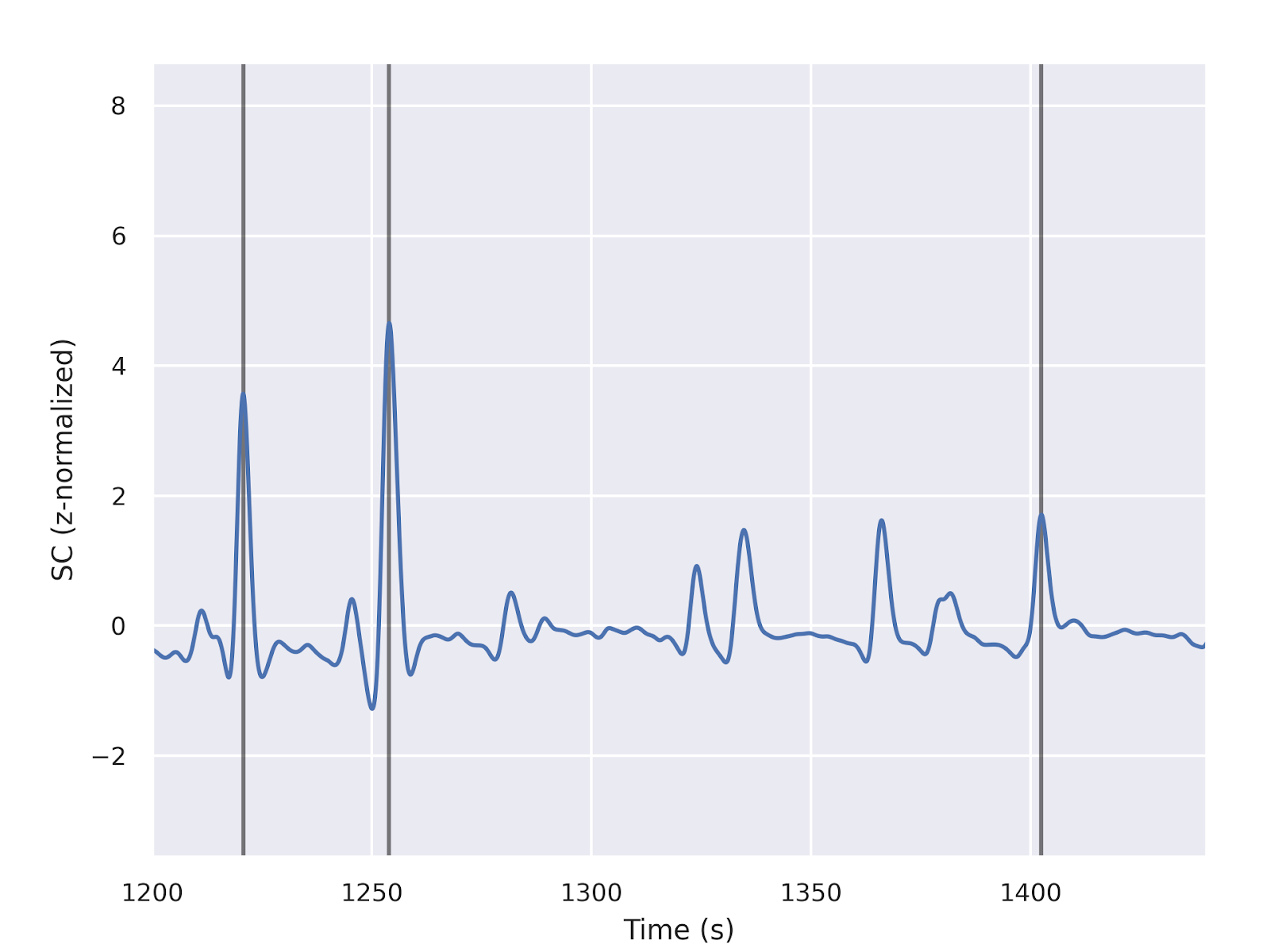
**

**
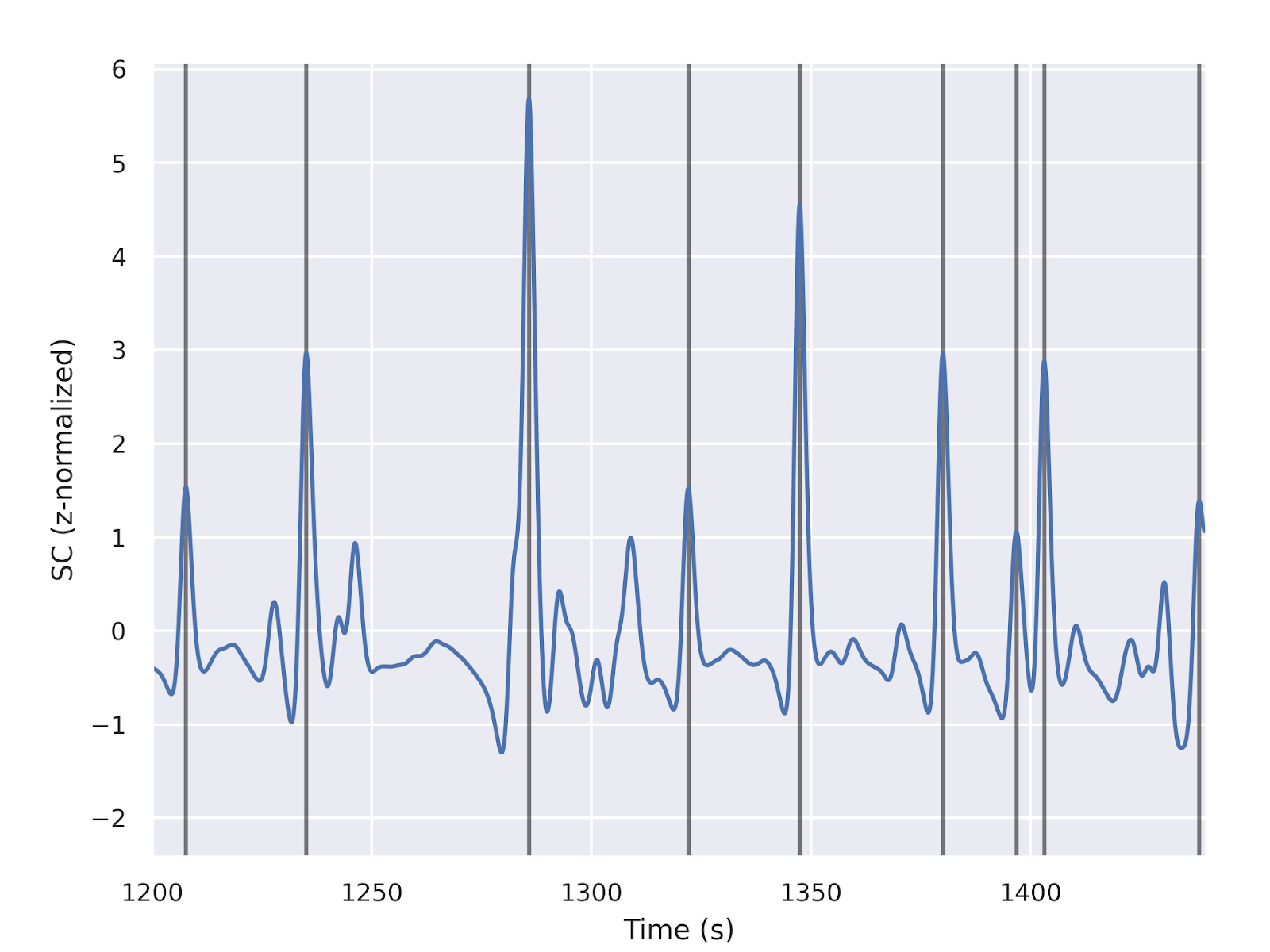
**

**
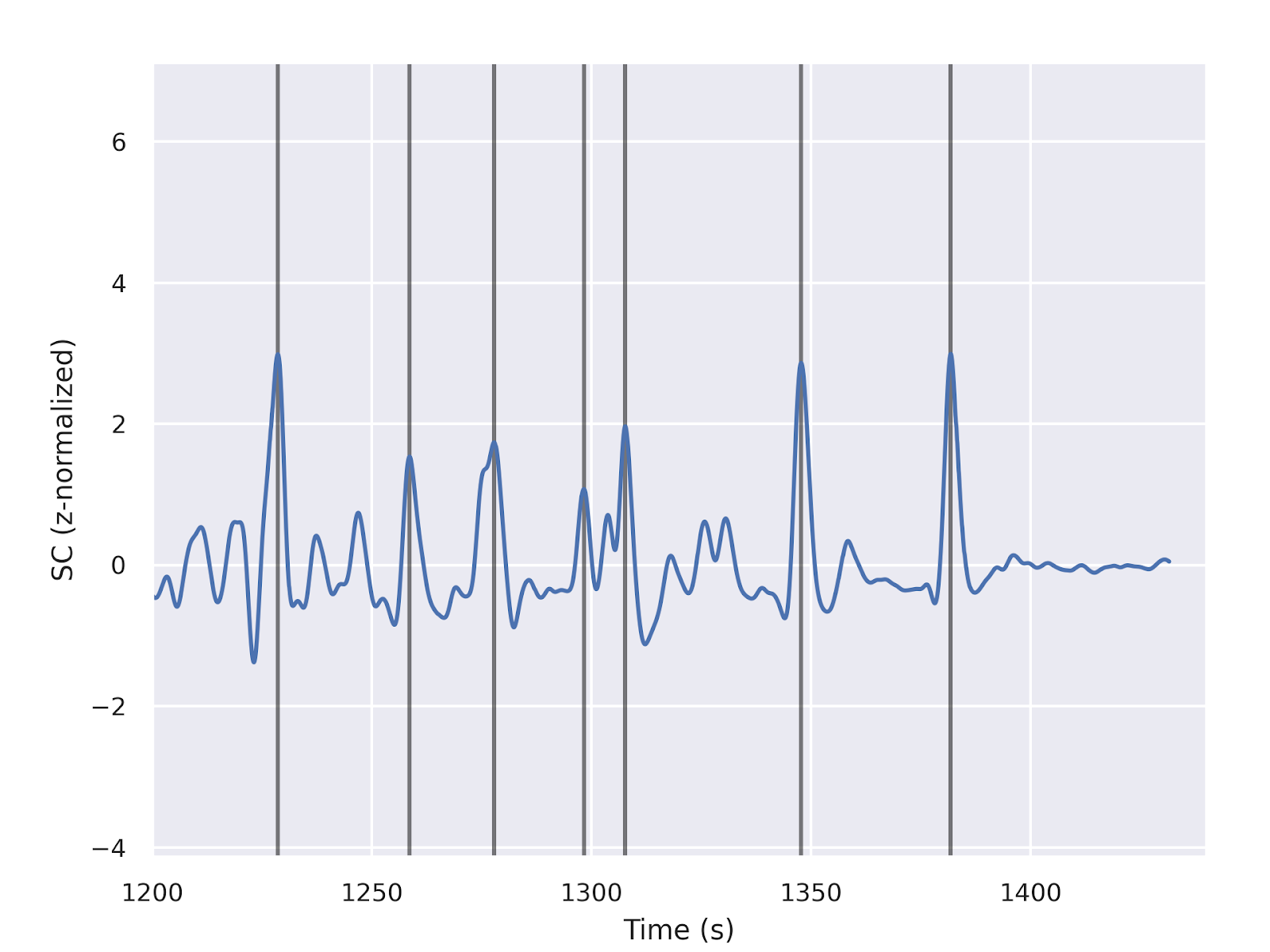
**
